# When Immunosuppression Fails: Cardiac Myxoma Mimicking Systemic Lupus Erythematosus Flare With a Diagnostic Delay

**DOI:** 10.7759/cureus.99337

**Published:** 2025-12-15

**Authors:** Gurdarshan Singh, Jaspreet Kaur

**Affiliations:** 1 Cardiology, Patiala Heart Institute and Multispeciality Hospital, Patiala, IND; 2 Cardiology, Sawai Man Singh Medical College, Jaipur, IND; 3 Medicine, Government Medical College, Patiala, Patiala, IND; 4 Pathology and Laboratory Medicine, Civil Hospital Samana, Patiala, IND; 5 Pathology, Government Medical College, Patiala, Patiala, IND

**Keywords:** anchoring bias, cardiac myxoma, diagnostic error, interleukin-6, syncope, systemic lupus erythematosus

## Abstract

A 36-year-old woman with well-controlled systemic lupus erythematosus (SLE) and prior pulmonary tuberculosis (TB) developed fever, weight loss, anemia (hemoglobin 7.6 g/dL), thrombocytopenia (78,000/mm³), and elevated inflammatory markers (ESR 89 mm/hr, CRP 67 mg/L), with symptom onset five months earlier (Month 0). Complement levels were normal, anti-dsDNA was negative, ANA remained unchanged, and she had no new rash, serositis, or renal involvement. Notably, she had no cardiac symptoms or abnormal findings on examination at that time. During the first month (Month 1), she was presumptively diagnosed with a clinically active but serologically quiescent SLE flare, based on fever, cytopenias, and elevated inflammatory markers in the context of her established SLE, despite normal complement levels and negative anti-dsDNA. Over the next three months (Months 1-3), infections with common bacterial, viral, and fungal pathogens were systematically excluded and typical causes of cytopenias were evaluated. During this period, she received escalating immunosuppression with corticosteroids, mycophenolate, and cyclophosphamide. Initial mild symptom relief was observed but waned within 2-3 weeks, and after three months, both symptoms and objective markers showed minimal improvement: ESR and CRP remained elevated, while hemoglobin and platelet counts remained essentially unchanged. At month four (Month 4), persistent fever despite immunosuppression raised concern for TB reactivation; empirical four-drug antitubercular therapy was briefly initiated but complicated by hepatotoxicity requiring cessation. TB cultures returned negative. At month five (Month 5), syncope prompted echocardiography, revealing a large (3.9×3.2 cm) pedunculated left atrial myxoma attached to the interatrial septum, prolapsing through the mitral valve during diastole. Cardiac MRI confirmed the diagnosis. Interleukin-6 was markedly elevated (107 pg/mL, normal <7). Following surgical excision, complete symptom resolution occurred with normalization of cytopenias by three months, a progressive decline in inflammatory markers, and IL-6 reduction to 12 pg/mL by six months. Immunosuppression was de-escalated to hydroxychloroquine alone with no recurrence at 14-month follow-up. This case illustrates diagnostic delay resulting from cognitive biases, including anchoring on her established SLE diagnosis and confirmation bias from partial steroid response, while highlighting underappreciated clinical red flags. Echocardiography was probably not performed during the course of the disease, given the absence of cardiac symptoms or abnormal findings on examination, and because the systemic symptoms were attributed to inflammatory causes. Critical underappreciated features included seronegative inflammation with normal complements and inadequate response to immunosuppression-atypical for genuine lupus flares. Inadequate treatment response within 4-6 weeks should trigger diagnostic reassessment before escalation. Syncope or orthostatic symptoms in systemic illness mandate urgent echocardiography. Cardiac myxomas produce IL-6 in the majority of patients (frequency >75%), and systemic constitutional signs are observed in a substantial proportion, though not uniformly. This can produce IL-6-mediated inflammation indistinguishable from autoimmune flares.

## Introduction

Cardiac myxoma is the most common benign primary cardiac tumor, accounting for around half to three‑quarters of benign cardiac tumors. It typically presents in middle‑aged adults (mean age ~50-51 years) with a female predominance (64.3%). Most arise in the left atrium (85.3%), often as pedunculated masses [1]. They may cause obstruction, embolic events, or systemic symptoms, though many are incidentally detected, contributing to diagnostic delay. Despite being the most common, they pose diagnostic challenges due to varying clinical presentation [2], particularly in patients with autoimmune disease. These benign neoplasms secrete interleukin-6 (IL-6), producing constitutional symptoms, cytopenias, and elevated inflammatory markers [3] that can mimic systemic lupus erythematosus (SLE) flares or infections like tuberculosis (TB). Typical SLE flares often present with fever, arthralgia or arthritis, rash, serositis, nephritis, cytopenias, or characteristic serologic changes such as low complement or elevated anti-dsDNA. Although constitutional symptoms and systemic inflammation occur in a substantial fraction of myxoma patients (variously reported between ~20 and 60%), there are no large studies quantifying how often they are misdiagnosed, especially in patients with pre‑existing autoimmune disease. In SLE, heightened cardiovascular morbidity [4] can lead clinicians to attribute new symptoms to the existing disease rather than structural pathology, a cognitive bias that can delay recognition of treatable conditions [5]. While myxomas mimicking autoimmune syndromes have been reported [6], few describe the reasoning errors underlying diagnostic delay during active immunosuppression. We report a patient treated for presumed lupus flare and later TB over five months before syncope led to echocardiographic detection of a large left atrial myxoma. Diagnostic delay in such cases often reflects specific cognitive biases. Anchoring on a patient’s established autoimmune diagnosis and premature closure after partial symptomatic improvement are well-described contributors to misdiagnosis in complex inflammatory presentations. Structural cardiac disease is under-recognized in immunosuppressed patients because systemic symptoms are usually attributed to autoimmune activity or opportunistic infection rather than mechanical causes. This case underscores how structural cardiac disease can masquerade as autoimmune activity and highlights the need for timely diagnostic reassessment when therapy fails to yield expected improvement.

## Case presentation

A 36-year-old woman with an 11-year history of well-controlled SLE on hydroxychloroquine 400 mg daily was referred with two syncopal episodes within 72 hours, following five months of progressive constitutional symptoms despite escalating immunosuppression at the referring facility. At the time of her initial SLE diagnosis, she had mild arthralgias, intermittent low-grade fever, weight loss, photosensitive rash, and fatigue, with no major organ involvement. Previous lupus flares (seven years back and five years back) had been mild, manifesting as fever, rash, and arthralgia, and had responded to short steroid courses. She had no prior renal, serosal, or hematologic involvement. Three years earlier, she had completed antitubercular therapy for culture-proven pulmonary tuberculosis (TB) with a cure. Clinical details from the preceding five months were obtained from referral records and laboratory data.

Five months prior (Month 0), she developed intermittent fever (38-39°C) and had anorexia, 5-kg weight loss, and mild arthralgia without rash, photosensitivity, or serositis. Labs showed normocytic anemia (hemoglobin 7.6 g/dL), thrombocytopenia (platelet count 78,000/mm³), elevated ESR (89 mm/hr) and CRP (67 mg/L), with normal leukocytes and renal function. Complement levels were normal (C3 118 mg/dL, C4 28 mg/dL), anti-dsDNA negative, and ANA unchanged from baseline (titres >1:320, homogeneous pattern). HRCT chest revealed healed right-upper-lobe fibronodular scarring, consistent with prior treated TB, with no evidence of active disease.

At Month 1, based on fever, cytopenias, and elevated inflammatory markers in the patient with established SLE, she was diagnosed with a clinically active but serologically quiescent SLE flare and treated with intravenous methylprednisolone pulse (methylprednisolone 500 mg IV once daily for three days) followed by oral steroids. After a transient improvement, the fever recurred on taper. Across the subsequent three months of treatment, usual causes of cytopenias were considered and deemed unlikely from clinical and laboratory assessment. Repeated blood and urine cultures, viral serologies, and fungal screening were negative. At three months, repeat tests showed persistently high ESR and CRP with normal complement levels. During the first three months (Month 1 to Month 3), diagnosis of SLE flare was favored because symptoms partially improved with steroid escalation initially, whereas infectious and malignant causes remained unsubstantiated at the time. Azathioprine was started (50 mg PO once daily), then switched to mycophenolate (500 mg PO twice daily) due to cytopenia, and later escalated to monthly cyclophosphamide (0.75 g IV, given single dose only), but fever and malaise persisted. Referring-facility records documented no earlier murmurs, orthostatic changes, or peripheral cardiac signs, reducing suspicion for structural heart disease.

At four months (Month 4), persistent fever despite immunosuppression raised concern for TB reactivation. Sputum AFB and GeneXpert were negative. Given high clinical suspicion in the heavily immunosuppressed patient with prior TB, persistent fever, and radiographic scarring, empirical antitubercular therapy was initiated pending cultures. Within two weeks, she developed hepatotoxicity (ALT 387, AST 312 U/L) requiring cessation of ATT. Liver function tests improved after cessation of therapy, confirming that the hepatotoxicity was attributable to ATT. Mycobacterium cultures later returned negative.

At five months (Month 5), labs showed minimal improvement (Hb 8.1 g/dL, platelets 82,000/mm³, ESR 76, CRP 60 mg/L; see Table 1). Cognitive bias toward systemic disease meant echocardiography was not considered, likely due to focus on systemic causes. She developed exertional dyspnea, orthostatic light-headedness, and two syncopal episodes, leading to referral for further evaluation.

**Table 1:** Laboratory parameters during the five-month treatment course at the referring facility and on presentation. Month zero to month five data as received from records of referring facility

Parameter	Month 0, Referring Facility	Month 1, Referring Facility	Month 4, Referring Facility	Month 5, Referring Facility	At Presentation at Our Center	Reference Range
							Hemoglobin	7.6	8.9	8.6	8.1	8	12–15 g/dL
White cell count	5,200	7800	11000	6,700	7200	4,000–11,000/mm³
Platelets	78,000	96000	85000	82,000	81000	150,000–400,000/mm³
ESR	89	59	72	76	82	0–20 mm/hr
CRP	67	61	53	60	72	<5 mg/L
Complement C3	118	98	Not done	112	123	90–180 mg/dL
Complement C4	28	22	Not done	29	24	10–40 mg/dL
Anti-dsDNA	12	15	Not done	Not done	11	<30 IU/mL (negative)
ANA	Positive, (titre >1:320,homogeneous pattern)	Not done	Not done	Not done	Positive (unchanged from baseline)	Negative (Titres <1:40)
Creatinine	0.9	1	1.1	0.8	0.9	0.6–1.2 mg/dL
BUN	11	15	13	12	14	7-20 mg/dL
Serum Bilirubin	0.9	1.1	2.4	1.4	1.2	0.1-1.2 mg/dL
AST	36	35	312	57	51	7-40 U/L
ALT	42	36	387	64	62	8-45 U/L
Serum alkaline phosphatase	113	124	143	135	141	44-150 IU/L
IL-6 levels	Not done	Not done	Not done	Not done	107	<7 pg/mL

Diagnostic breakthrough (month 5)

On admission, she appeared chronically ill with pallor. BP was 118/74 mmHg supine (100/60 standing) and HR 92 bpm supine (106 bpm standing), indicating orthostatic changes. Cardiac exam revealed a low-pitched early diastolic sound and a mid-diastolic murmur at the apex, more prominent in sitting posture. Syncope and orthostatic symptoms in a systemic inflammatory context prompted immediate echocardiography instead of further immunosuppression.

Echocardiographic findings

**Figure 1:**
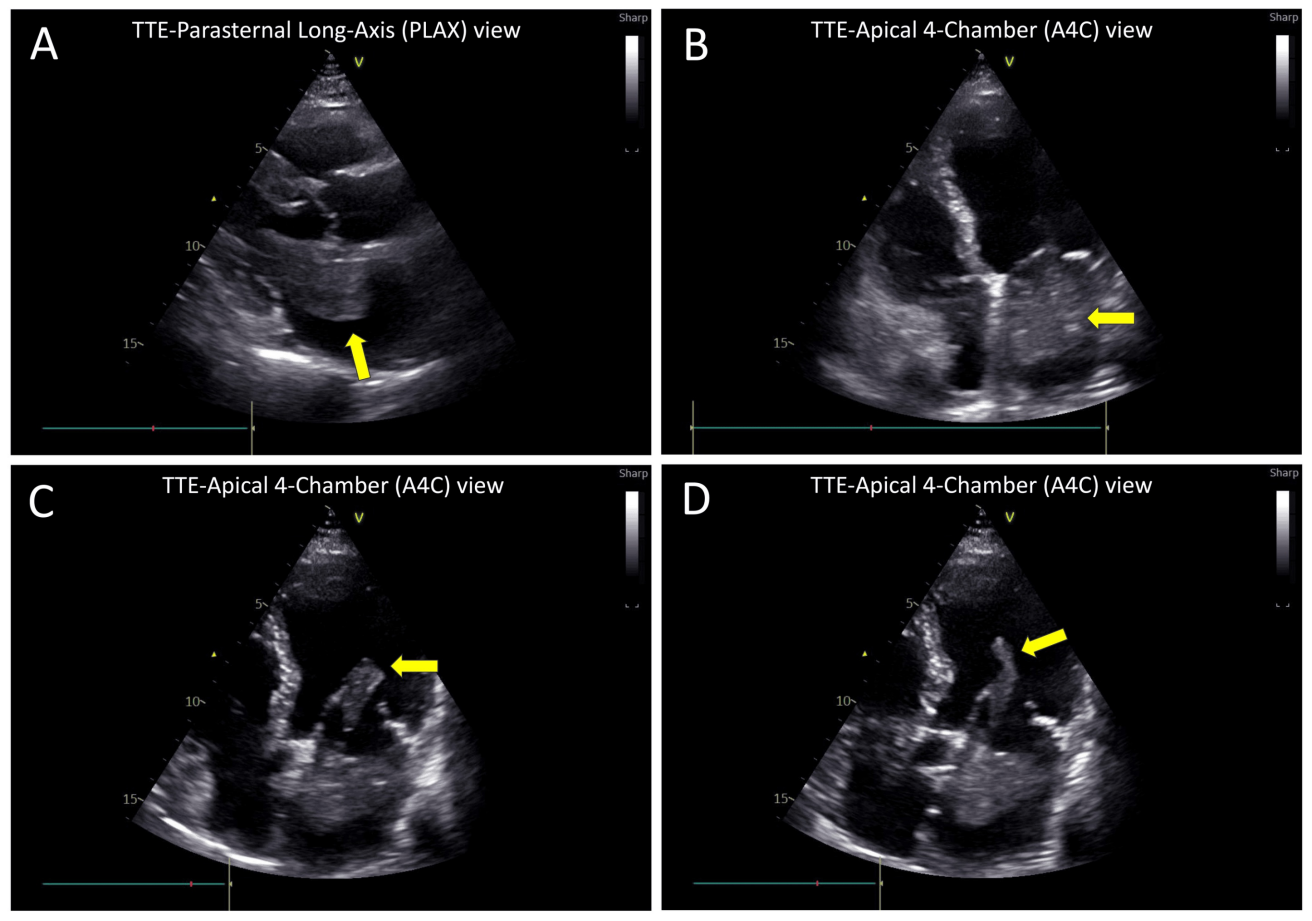
Transthoracic echocardiography (TTE) TTE showing a large mass in the left atrium (LA) abutting the mitral valve (MV). Panels A-D: (A) Parasternal long-axis view, (B) Apical four-chamber view in systole showing a round mass in the LA attached to the interatrial septum, positioned near the MV, (C) Apical 4-chamber view in early diastole showing finger-like pedunculation attached to the LA mass, prolapsing through the MV, almost completely obstructing left ventricular inflow. (D) Apical four-chamber view in late diastole showing pedunculation projecting further into the left ventricle with partial obstruction of inflow.

Transthoracic echocardiography demonstrated normal biventricular function (LVEF 63%), mild aortic regurgitation and no pericardial effusion. A large, freely mobile, round-shaped mass measuring 3.9 × 3.2 cm was identified in the left atrium (LA), attached to the interatrial septum near the fossa ovalis (Figures 1A, 1B). The mass exhibited a finger-like pedunculated attachment (3.8 cm long), which prolapsed through the mitral valve (MV) into the left ventricle (LV) during diastole, partially obstructing LV inflow (Figures 1C, 1D) (Video 1 and Video 2). The echocardiographic features were most consistent with a myxoma. Other differentials included thrombus, rare primary cardiac tumors such as lipoma or sarcoma, and, less likely, papillary fibroelastoma.

**Video 1:** Transthoracic echocardiography (TTE) TTE apical four-chamber view showing a large mobile mass in the left atrium (LA) with a long finger-like projection prolapsing through the mitral valve (MV), which is partially obstructing inflow.

**Video 2:** Transthoracic echocardiography (TTE) TTE parasternal long-axis view showing a large freely mobile mass in the left atrium (LA) abutting the mitral valve (MV).

**Figure 2:**
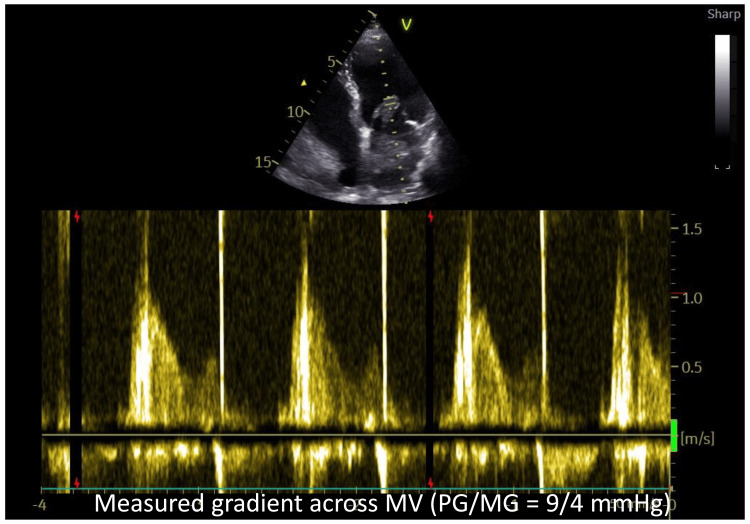
Continuous-wave (CW) Doppler interrogation across the mitral valve The CW Doppler tracing across MV demonstrates a peak gradient of 9 mmHg and mean gradient of 4 mmHg, indicating hemodynamically non-significant obstruction at rest despite the large tumor size.

Calculated transmitral pressure gradient at rest was not significant (peak/mean gradient=9/4 mmHg) (Figure 2).

Cardiac MRI

**Figure 3:**
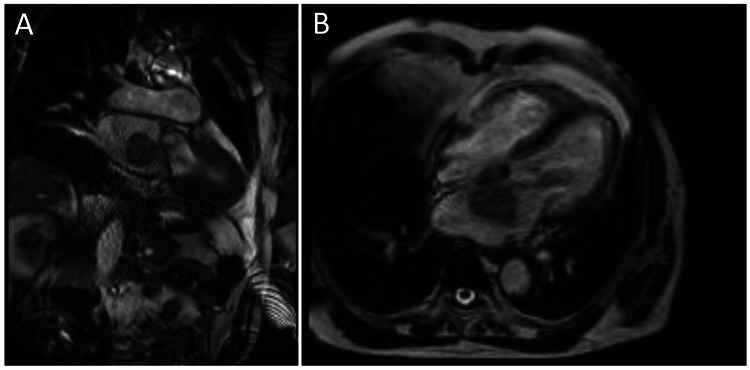
Cardiac MRI showing (A) 4.2 × 3.5 × 3.6 cm heterogeneous mass in the left atrium (LA) arising from the interatrial septum, with characteristics consistent with myxoma. (B) Mass is seen with an attached finger-like projection prolapsing through the mitral valve.

Cardiac MRI using a balanced steady-state free precession (bSSFP; Siemens TRUFI) cine sequence demonstrated a large pedunculated left atrial mass (4.2 × 3.5 × 3.6 cm) attached near the fossa ovalis prolapsing toward the mitral orifice without invading adjacent structures. Mass was intermediate T1 and high T2 signal with heterogeneous post-contrast enhancement, differentiating it from thrombus and lipoma, with findings most consistent with myxoma. Biventricular morphology and systolic function were preserved (Figure 3).

Surgical management and outcome

She underwent urgent median sternotomy and cardiopulmonary bypass for complete excision of the mass with attached interatrial septum, repaired with a pericardial patch. Intraoperatively, the tumor appeared friable with potential embolic risk, supporting the urgency of surgical excision. Histopathology confirmed myxoma with satellite and polygonal cells in abundant myxoid stroma. IL-6 levels on arrival were 107 pg/ml decreasing gradually postoperatively. Patient’s symptoms started improving within two weeks, with complete recovery over the next six months (Table 2). No residual structural cardiac abnormalities were identified, and the patient did not experience any embolic events during the clinical course or subsequent follow-up.

**Table 2:** Postoperative vital clinical and laboratory parameters Timeline of clinical recovery and laboratory response after surgical excision of left atrial myxoma.

Timepoint	Clinical Status	Laboratory Values
Week 2	Fever resolved, appetite returning, exercise intolerance post-surgery	Hb-9.1 g/dL; ESR-45 mm/hr; CRP-42 mg/L; Platelets-105,000/mm³
Month 1	Complete symptom resolution, started gaining weight, improved exercise tolerance	Hb-9.8 g/dL; ESR-36 mm/hr; CRP-18 mg/L; IL-6- 54 pg/mL; Platelets-126,000/mm³
Month 3	Gaining weight, normal activity and exercise tolerance	Hb 11.4 g/dL; ESR-18 mm/hr; CRP-4.7 mg/L Platelets 162,000/mm³;
Month 6	Recovered completely, asymptomatic, had gained 8 Kg weight	All parameters normalized; IL-6 12 pg/mL

Immunosuppression was de-escalated postoperatively. Corticosteroids were tapered gradually, and the patient did not exhibit clinical or biochemical evidence of adrenal insufficiency. She remains on hydroxychloroquine alone with no evidence of SLE activity at 14-month follow-up. Serial echocardiography at six and 12 months shows no recurrence. Given the isolated lesion and lack of syndromic features, recurrence risk was considered low, and screening for Carney complex was not done.

## Discussion

The clinical challenge of overlapping syndromes

This case exemplifies the diagnostic challenge posed by structural cardiac lesions that produce systemic inflammatory syndromes mimicking autoimmune disease. Cardiac myxomas secrete IL-6 and other inflammatory mediators, resulting in constitutional symptoms in almost half of patients [3]. But it’s not clear how often IL-6-mediated systemic inflammation in myxoma can be mistaken for autoimmune flares. The clinical presentation, fever, weight loss, anemia, thrombocytopenia, and elevated acute-phase reactants, overlaps substantially with SLE flares, making differentiation challenging without cardiac imaging.

While prior case reports have documented myxomas mimicking autoimmune syndromes [7], this case is notable for the five-month diagnostic delay despite progressive treatment failure. The key question is what features should have prompted earlier reconsideration.

Diagnostic red flags that were present but underappreciated

Two critical features were present but underappreciated: First, the combination of seronegative inflammation, normal complement levels, negative anti-dsDNA, and absence of typical SLE manifestations over five months was atypical for a genuine lupus flare. Clinically active but serologically quiescent SLE flare, clinical manifestations without complement consumption or anti-dsDNA elevation, though relatively uncommon, are estimated to occur in roughly 10-20% of SLE flares, but their occurrence explains why the initial diagnosis of a lupus flare was clinically plausible, despite atypical laboratory features. Second, despite high-dose corticosteroids, additional immunosuppressants, and a brief empirical trial of antitubercular therapy that was discontinued due to hepatotoxicity, objective markers showed minimal improvement. In genuine SLE flares and TB, appropriate therapy typically produces substantial improvement within 4-6 weeks. In contrast, cytokine-driven tumors such as cardiac myxomas may produce persistent IL-6-mediated inflammation unresponsive to steroids or cytotoxic agents, leading to sustained fever, cytopenias, and elevated inflammatory markers despite aggressive therapy. This differential response can serve as an early clinical clue prompting reconsideration of structural, neoplastic, infectious, drug-induced, or hematologic causes.

Factors contributing to diagnostic delay at the referring facility

Anchoring on established diagnosis: The patient's 11-year SLE history created a powerful explanatory framework, with prior flares showing similar systemic features. Partial response providing false reassurance: Initial fever reduction and modest hemoglobin improvement (7.6 → 8.9 g/dL) suggested correct diagnosis with inadequate treatment, rather than wrong diagnosis. This likely reflected non-specific anti-inflammatory effects of corticosteroids on cytokine-mediated symptoms rather than treatment of underlying pathology, misleading the clinicians. Premature closure within a restricted framework: When immunosuppression failed, the diagnostic pivot to TB was clinically reasonable given the patient's history. In drug-sensitive TB, clinical and laboratory improvement is typically observed within 2-6 weeks of therapy, with fever resolution often occurring within the first 2-4 weeks; however, in this case, the patient received only a brief empirical four-drug trial that was discontinued early due to hepatotoxicity, so no meaningful interpretation regarding TB treatment response could be made. But persistent systemic inflammation despite normal complements should have prompted consideration of other infections (bacterial, viral, fungal), malignancy (hematologic, solid tumors), structural cardiac lesions, and drug-induced or marrow-mediated cytopenias. However, this represented autoimmune-to-infectious reasoning without systematic consideration of structural or neoplastic categories. Absence of cardiac symptoms until late: Without syncope or positional symptoms, cardiac evaluation was not prioritized. Echocardiography is not routine for presumed lupus flares or fever of unknown origin without cardiac manifestations. There was a lack of structured reassessment protocols; no systematic reassessment was triggered despite objective treatment failure. Modern diagnostic frameworks emphasize iterative reasoning with continuous probability updating based on treatment response, but implementation remains inconsistent.

The turning point: syncope as a de-anchoring event

Syncope and orthostatic presyncope were transformative symptoms; they introduced a clear mechanical component not attributable to systemic inflammation alone. The recognition that syncope in the context of chronic illness requires structural cardiac evaluation was the critical clinical decision that led to diagnosis. In patients with persistent systemic inflammation, especially when fevers, cytopenias, or inflammatory markers do not respond within 4-6 weeks, early cardiology consultation and low-threshold echocardiography may facilitate the timely detection of structural lesions such as cardiac myxomas.

The role of IL-6 in diagnosis and monitoring

Cardiac myxomas produce IL-6 in the majority of patients (frequency >75%. IL-6 levels were not measured at the referring facility. At presentation to our hospital, IL-6 was markedly raised (107 pg/ml). IL-6 decline following surgical removal of the tumor supported a non-autoimmune source of inflammation. While IL‑6 elevation is non-specific-occurring in active lupus, infections, and malignancies [8], its dramatic decline supported tumor-derived cytokine production (Table 2). Serial IL-6 measurements postoperatively primarily correlated with symptom resolution but were not used to guide further management. Whether routine IL-6 measurement could expedite diagnosis remains uncertain, as discriminatory thresholds have not been established [9].

While “anchoring bias” is often cited in diagnostic errors [5], this case illustrates a more complex issue. Individual-level errors included anchoring on the established SLE diagnosis and confirmation bias from partial steroid response. System-level contributors included the absence of structured reassessment protocols, delayed multidisciplinary consultation, and underutilization of echocardiography in prolonged systemic inflammation. The initial diagnosis-seronegative lupus flare in a patient with established SLE-was reasonable. The critical error occurred during follow-up when inadequate treatment response was misinterpreted, emphasizing the need for iterative diagnostic reasoning [10]. This reflects systems-level errors: confirmation bias (partial steroid response seen as diagnostic confirmation), sequential anchoring (shifting from lupus to TB without broadening the differential), and underuse of treatment response as a diagnostic tool. Modern frameworks stress iterative diagnosis, continuously updating probabilities based on new data, including response to therapy [11]. The failure thus lay in subsequent reasoning cycles, not the initial formulation.

## Conclusions

Cardiac myxomas can produce IL-6-mediated systemic inflammation mimicking autoimmune flares, which can lead to diagnostic delay in patients with established SLE. In SLE, guideline-directed therapy response is judged by improvement in fever, cytopenias, inflammatory markers, and organ-specific parameters within the expected timeframe. While not absolute, a lack of meaningful improvement over approximately 4-6 weeks in SLE generally serves as a useful clinical signal to reassess the diagnosis. When systemic inflammation persists despite normal complement levels and negative anti-dsDNA, alternative diagnoses to consider include infections, hematologic malignancies, drug-induced cytopenias, and structural or neoplastic causes such as cardiac tumors. While elevated IL-6 may support consideration of neoplastic or structural causes of systemic inflammation, it remains a non-specific cytokine and should not be used as a stand-alone diagnostic marker. This case underscores that inadequate response to appropriate therapy for the presumed diagnosis should prompt diagnostic reassessment before treatment escalation. Orthostatic symptoms, presyncope or syncope in patients with unexplained systemic illness mandate urgent cardiac imaging and echocardiography. Clinicians must implement structured reassessment protocols when treatment fails to produce expected outcomes, moving beyond initial diagnostic formulations to consider alternative structural pathology.
